# *ADGRL3*, *FGF1* and *DRD4*: Linkage and Association with Working Memory and Perceptual Organization Candidate Endophenotypes in ADHD

**DOI:** 10.3390/brainsci11070854

**Published:** 2021-06-26

**Authors:** Martha L. Cervantes-Henriquez, Johan E. Acosta-López, Mostapha Ahmad, Manuel Sánchez-Rojas, Giomar Jiménez-Figueroa, Wilmar Pineda-Alhucema, Martha L. Martinez-Banfi, Luz M. Noguera-Machacón, Elsy Mejía-Segura, Moisés De La Hoz, Mauricio Arcos-Holzinger, David A. Pineda, Pedro J. Puentes-Rozo, Mauricio Arcos-Burgos, Jorge I. Vélez

**Affiliations:** 1 Facultad de Ciencias Jurídicas y Sociales, Universidad Simón Bolívar, Barranquilla 080005, Colombia; jacosta@unisimonbolivar.edu.co (J.E.A.-L.); mostapha.ahmad@unisimonbolivar.edu.co (M.A.); sanchezr@unisimonbolivar.edu.co (M.S.-R.); gdjimenez@unisimonbolivar.edu.co (G.J.-F.); wpineda1@unisimonbolivar.edu.co (W.P.-A.); mmartinez108@unisimonbolivar.edu.co (M.L.M.-B.); lnoguera1@unisimonbolivar.edu.co (L.M.N.-M.); emejia18@unisimonbolivar.edu.co (E.M.-S.); mdelahoz48@unisimonbolivar.edu.co (M.D.L.H.); 2 Universidad del Norte, Barranquilla 081007, Colombia; 3 Grupo de Investigación en Psiquiatría (GIPSI), Departamento de Psiquiatría, Instituto de Investigaciones Mxdicas, Facultad de Medicina, Universidad de Antioquia, Medellin 050010, Colombia; oscararcos98@gmail.com (M.A.-H.); mauricio.arcos@udea.edu.co (M.A.-B.); 4 Grupo de Neuropsicología y Conducta, Universidad de San Buenaventura, Medellín 050010, Colombia; david.pineda@usbmed.edu.co; 5 Grupo de Neurociencias del Caribe, Universidad del Atlántico, Barranquilla 081001, Colombia; ppuentes1@unisimonbolivar.edu.co

**Keywords:** Caribbean community, ADHD, *FGF1*, DRD4, ADGRL3, endophenotypes, perceptual organization, working memory

## Abstract

Attention deficit hyperactivity disorder (ADHD) is a highly heritable neurobehavioral disorder that affects children worldwide, with detrimental long-term consequences in affected individuals. ADHD-affected patients display visual–motor and visuospatial abilities and skills that depart from those exhibited by non-affected individuals and struggle with perceptual organization, which might partially explain impulsive responses. Endophenotypes (quantifiable or dimensional constructs that are closely related to the root cause of the disease) might provide a more powerful and objective framework for dissecting the underlying neurobiology of ADHD than that of categories offered by the syndromic classification. In here, we explore the potential presence of the linkage and association of single-nucleotide polymorphisms (SNPs), harbored in genes implicated in the etiology of ADHD (*ADGRL3*, *DRD4*, and *FGF1*), with cognitive endophenotypes related to working memory and perceptual organization in 113 nuclear families. These families were ascertained from a geographical area of the Caribbean coast, in the north of Colombia, where the community is characterized by its ethnic diversity and differential gene pool. We found a significant association and linkage of markers *ADGRL3*-rs1565902, *DRD4*-rs916457 and *FGF1*-rs2282794 to neuropsychological tasks outlining working memory and perceptual organization such as performance in the digits forward and backward, arithmetic, similarities, the completion of figures and the assembly of objects. Our results provide strong support to understand ADHD as a combination of working memory and perceptual organization deficits and highlight the importance of the genetic background shaping the neurobiology, clinical complexity, and physiopathology of ADHD. Further, this study supplements new information regarding an ethnically diverse community with a vast African American contribution, where ADHD studies are scarce.

## 1. Introduction

Attention deficit hyperactivity disorder (ADHD) is a neurobehavioral disorder that affects 8 to 18% of the population [1,2,3,4]. Inattention, hyperactivity and impulsivity symptoms are more frequent in ADHD individuals than in children and adolescents of the same age and developmental level, with ADHD symptoms persisting into adulthood in 40–50% of cases [5]. Heritability estimates indicate that the genetic factors explain up to 75–95% of symptoms’ variability of the disorder [6,7].

Endophenotypes are quantifiable or dimensional constructs that are closely related to the final physiopathological cause of the disease [8,9,10,11]. Endophenotypes, or the extreme presentation of these traits: (i) occur more frequently in individuals with the disease, (ii) co-segregate in families, and/or (iii) manifest in individuals with variable expressivity and/or variable penetrance depending upon whether the disease is present [10,11]. Thus, endophenotypes are seen as a *proxy* to the actual phenotype as they connect behavioral symptoms with the well-understood illness associated with known genetic causes [8,12,13,14,15]. In ADHD, endophenotypes have allowed the definition of potential neurobiological markers for detecting ADHD susceptibility loci [10,13,16,17,18]. Due to their continuous nature, endophenotypes are more powerful, statistically speaking, to uncover genetic variants associated with the disease and, therefore, the sample size required for its detection is smaller if compared to the sample size needed when dealing with binary traits [18,19].

ADHD individuals lack visual–motor skills and visuospatial ability [16,20] and show alterations in the perception and processing of temporal information [21,22,23], difficulties in scheduling behaviors and tolerating waiting [24], deficiencies in perceptual organization, and deficiencies in the measurement of sensory–motor function, stimulus processing (i.e., auditory, visual, tactile and kinesthetic), stimulus–response association, time to produce correct responses, speed–efficiency balance, decision making and response programming [25], which lead to impulsive responses [26,27]. Furthermore, ADHD-affected individuals show a myriad of symptoms in different dimensions, and evident difficulties in sensory modalities and perceptual organization [28], working memory [29] and attention use in tasks based on visuospatial aspects [30,31,32,33], which are important constructs for spatial and verbal processing in higher order tasks [34] and are crucial for the identification and recognition of objects and surfaces in the environment [35]. Such difficulties coexist with cognitive and behavioral aspects [36].

Changes in pupil size and diameter may reflect increased mental and/or cognitive effort [37,38,39], and this is one of the mechanisms that reveals an underlying neuronal action [40]. In ADHD, pupil size is associated with possible alterations at the visual perception level [41,42,43] and related to attentional and behavioral processes (i.e., reaction times) as well as to the perceptive identification through eye movements and duration of fixation [44,45]. Thus, deficits in sensory activation ability may explain some of the central deficits found in ADHD [46,47,48].

We recently identified ADHD cognitive endophenotypes related to working memory and perceptual organization in nuclear families segregating ADHD [49] in a Caribbean community with one of the largest African admixtures in Colombia and South America [50,51,52]. Now, in this study, we explore whether genetic variation is a major component underpinning the biological basis of these traits. Single nucleotide polymorphisms (SNPs) harbored in genes previously associated with ADHD in our family-based cohort were tested for linkage and association with endophenotypes of working memory and perceptual organization. Our overarching hypothesis is that genetic variation implicated in conferring susceptibility to ADHD is also associated with working memory and perceptual organization, which in turn may help to better understand the underlying mechanisms shaping the complexity of multisensorial alterations in ADHD.

## 2. Materials and Methods

### 2.1. Subjects

We recruited and clinically characterized 386 individuals (218 (56.5%) males, 168 (43.5%) females; 224 (58%) with ADHD, 162 (42%) controls) from 113 nuclear families with at least one ADHD proband. A total of 120 (31.1%) were children (6–11 years), 34 (8.8%) were adolescents (12–17 years) and 232 (60.1%) were adults (>17 years). All individuals were born in and inhabit the metropolitan area of Barranquilla, Colombia. No children or adults were treated with medication for ADHD at initial assessment. The full neurological, neuropsychological and psychological assessment, as well as demographic information, has been reported elsewhere [49,52,53]. Briefly, 408 individuals belonging to 120 nuclear families and ascertained from probands affected by ADHD initially participated in our clinical and genetic studies of ADHD. ADHD diagnosis was assessed in all individuals using behavioral [54,55,56] and psychopathological interviews, which include the structured Diagnostic Interview for Children and Adults (DICA) version IV [57]. This interview (1) considers the A criterion of the DSM-IV, (2) utilizes a systematic approach to collect clinical information about ADHD symptoms exhibited by an individual using a binary classification (0 = absent; 1 = present) system, and (3) has been extensively used by our group and others in genetic studies of ADHD [27,49,52,53,58,59,60]. ADHD symptom data were collected during the clinical assessment stage, where 11 schools were visited (seven of medium socio-economic stratum). Several meetings were held with teachers of children aged between 6 and 11 years old to explain the objective of the study. Teachers were asked to identify children about whom they had concerns that might affect their academic performance and/or behavior in the school environment. Parents or guardians were administered the Spanish version of the DICA-IV interview for parents (DICA-IV-P). As genetic data were not available for seven families from the original cohort, only 113 out of the 120 nuclear families were included in the present study. This study was approved by the Ethics Committee of Universidad Simón Bolívar, Barranquilla, Colombia (approval # 00032, 13 October 2011).

### 2.2. Endophenotypes

Based on the multidimensional clinical assessment, we recently identified that the digits forward and backward, arithmetic and similarities were candidate endophenotypes in Caribbean families segregating ADHD. These tasks have been related to the phonological loop of working memory (digit forward), central executive working memory (digit backward), and working memory episodic buffer (arithmetic and similarities) [61,62,63], which correspond to working memory components [32,64,65]. Furthermore, neuropsychological tasks such as the completion of figures and the assembly of objects, which would assess visual detail detection, spatial object orientation, and visuospatial problem-solving cognitive domains, constitute endophenotypes of working memory and perceptual organization based on a hierarchical model of functions [49,66,67,68] (Table 1). A machine learning algorithm including these endophenotypes predicts ADHD diagnosis with 81.5% accuracy (95% confidence interval (CI) = 77.5–85.0) [49].

### 2.3. DNA Extraction and Genotyping

DNA extraction and genotyping were performed as described elsewhere [52]. Briefly, genomic DNA was isolated from blood samples using the MasterPure^®^ DNA Purification Kit (Epicentre Biotechnologies, Chicago, IL, USA) according to the manufacturer’s protocol. DNA concentrations were measured using a NanoDrop™ 2000 spectrophotometer (Thermo Fisher Scientific, Waltham, MA, USA). Genotyping was performed at the University of Arizona Genetics Core using the multiplex Sequenom^®^ Technology on Agena Bioscience’s MassARRAY^®^ MALDI-TOF instrument. A total of 26 intronic SNPs were initially genotyped in our family-based cohort (Supplementary Materials Table S1) [52].

Allele and genotype frequencies were estimated using maximum likelihood. Mendelian errors and missing genotypes, common features in SNP-based genotyping, were detected and subsequently corrected with the methods available in Golden Helix’s^®^ SNP variation suite (SVS) 8.4.0 (Golden Helix, Inc. Bozeman, MT, USA; https://www.goldenhelix.com/, accessed on 19 December 2019). Golden Helix’s SVS is an integrated collection of analytic tools for managing, analyzing, and visualizing multifaceted genomic and phenotypic data. In this study, only SNPs harbored in genes previously reported to confer susceptibility to ADHD in our sample were selected to explore their association with ADHD endophenotypes of working memory and perceptual organization.

### 2.4. Family-Based Association Analysis

We used the family-based association test (FBAT) to assess linkage and association of SNPs with perceptual organization endophenotypes in our cohort, which includes complex family structures with multiple affected individuals and, in some cases, several probands, which introduces complex patterns of ascertainment. The FBAT provides a unified framework to generalize the transmission disequilibrium test [69,70] and accounts for different genetic models, sampling of family-based ascertainment designs, disease phenotypes, missing parents, and different null hypotheses [70]. Furthermore, FBAT screening methods are minimally affected by non-causal SNPs, and are robust against effects of population stratification and admixture, since the final decision is based on the FBAT statistic [71].

We used the implementation of the FBAT provided in the Pedigree-based association test (PBAT) module of Golden Helix’s SVS 8.4.0. The FBAT allows the testing of a combination of phenotypes (as a group) and genotypes that have the highest power by those predicted from the parents’ genotypes. As age and gender are known to impact ADHD susceptibility, both variables were included as ADHD covariates under the hypothesis of no linkage and no association. Adding these covariates increases the FBAT power substantially [72,73]. Additive, dominant, recessive and heterozygous advantage models of inheritance were explored to assess the association between SNPs and working memory and perceptual organization endophenotypes. Under an additive model, having 0, 1 or 2 copies of the major allele linearly increases/decreases the value of an endophenotype; under a dominant model, having at least one copy of the dominant allele increases/decreases the value of the endophenotype; and under a recessive model, having two copies of the minor allele increases/decreases the endophenotype versus having one or no copies. *P*-values from the FBAT were corrected for multiple testing using Bonferroni’s method [74,75].

## 3. Results

As multiple tests were applied in our genetic association analysis (i.e., five di-allelic markers, two covariates and four models of inheritance, resulting in a total of 5 × 2 × 4 = 40 tests), *p*-values were corrected using Bonferroni’s method. Table 2 shows the main results of the FBATs.

We found significant association and linkage of markers *ADGRL3*-rs1565902, *DRD4*-rs916457 and *FGF1*-rs2282794 to the performance on the arithmetic (T47), similarities (T48), figure completion (T49) and object assembly (T52) subtests after FDR correction.

Our results suggest that marker *ADGRL3*-rs1565902 is statistically significantly associated and linked to the performance in the arithmetic subtest under the additive model of inheritance (*p* = 0.014; Table 2). Similarly, we found evidence of linkage and association of marker *DRD4*-rs916457 with the performance in the figure completion subtest under the additive (*p* = 0.005) and dominant (*p* = 0.005) genetic models of inheritance (Table 2).

In addition, marker *FGF1*-rs2282794 was found to be associated with the performance in the similarities subtest under the additive (*p* = 0.004), dominant (*p* = 0.00019) and recessive (*p* = 0.00019) models of inheritance; with the performance in the figure under the dominant (*p* = 0.006) and recessive (*p* = 0.006) models of inheritance; and with the performance in the object assembly subtest under the heterozygous advantage model of inheritance (*p* = 0.005; Table 2).

## 4. Discussion

Genetic studies in ADHD have mainly focused on identifying associations with nuclear symptoms [76], with genes delineating visual–constructional skills at the perceptual organization level being identified in Caucasian and Asian populations [77]. In this study, we explore the association between SNPs and endophenotypes of working memory and perceptual organization [49] in 113 nuclear families from an understudied Caribbean community segregating ADHD and inhabiting the metropolitan area of Barranquilla, Colombia. The Colombian Caribbean region is the result of a racial admixture and the presence of a large African genetic component [50,51,78].

ADHD etiology is complex and strongly related to neurotransmitter pathways, i.e., dopamine [42]. Family-based and genome-wide association studies (GWAS) have identified single nucleotide polymorphisms (SNPs) in the *DRD4* gene associated with ADHD [43,79,80,81,82,83,84,85]. Furthermore, genetic variants in the *FGF1* [17,52] and *ADGRL3* (*LPHN3*) [17,52,58,59,83,86,87,88,89,90,91,92,93] genes are associated with ADHD and ADHD endophenotypes. Other endophenotype-associated genes include *DAT1*, *COMT*, *DBH*, *MAOA*, *DRD5*, *ADRA2A*, *GRIN2A*, *BDNF* and *TPH2* [94,95].

Here, we found that variants *DRDR4*-rs916457, *FGF1*-rs2282794 and *ADGRL3*-rs1565902 are associated with ADHD endophenotypes defined by the total digits, arithmetic and analogies, which evaluate working memory components [33,96], as well as incomplete figures and object assembly, which are related to perceptual organization (Table 2). In addition to allowing the identification of genes correlated with ADHD, linkage and association analyses of endophenotypes also allow the exploration of the underlying cognitive processes, such as perceptual organization [8,16,17], which is correlated with neuropsychological functioning during stimulus processing such as tracking and visual search tasks, and is widely used to examine automatic selective attention and stress [97,98].

Cortical activations differ between visual and auditory stimuli, with greater variability in the former [99]. In addition, the type of presentation seems to also influence the determination of response accuracy; these group differences suggest deficiencies in the basic timing mechanisms, such as neurophysiological resource failure, for ADHD [100]. Electrophysiological and imaging studies in ADHD show a generated alpha-lateralized modulation in the occipital and parietal cortex [101,102]. At the structural level, there is a correlation between the number of attentional failures and the volume of gray matter in the left occipital cortex [103,104]. Studies using event-related potentials indicate a significant involvement of the visual cortex in visual attention tasks [105,106,107]. Similarly, studies with functional magnetic resonance show a lower occipital activation in perceptive processes of visual attention in ADHD [108].

These deficiencies are a combination of cognitive control processes, as working memory components, and the perceptual integrative processes, among which are: visual coding, visual processing of the stimulus [109,110], sensory and eye pursuit movement, binocular tracking [111,112], perceptual attention profiles [113], perceptual variability, visual perception and representation, time of visual processing, visual–motor skills [114], resolution of visuospatial problems, self-reported deficiencies in perceptual function and abnormal perceptual experience [8,16,17,21,22,46,47,48,115,116,117,118]. Clinically abnormal perceptions affect visual memory, spatial relationships and sequential memory, suggesting specific patterns of altered visual perception [41,47,119,120]. This is mainly due to the fact that subcomponents of working memory are closely related to each other, as well as with other cognitive systems such as long-term memory (LTM), executive functions, attention and information processing speed [121]. Due to the complexity and heterogeneity of ADHD, such processes have been proposed as probable endophenotypes [122,123,124,125].

The receptor encoded by the *DRD4* gene is highly expressed in several key brain regions, including the prefrontal cortex, the medial temporal lobe, the hippocampus, the amygdala, and the hypothalamus [126,127,128,129,130], which are related to autoregulation deficits and the functional and structural integrity of the primary sensorial network, and regulates the efficiency of the central dopaminergic pathway involved in the post-synaptic action of dopamine [131]. It has been shown that DRD4 modulates the surrounding response of the center *off* ganglion cell, favoring spatial contrast sensitivity and color vision and enhancing the trophic function, retinal cell survival, and eye growth [132]. More recently, *DRD4* has been associated with the occurrence of the subject’s blink as an index of dopaminergic activity behavior [133]. Furthermore, *DRD4* is involved in perception and response to perception as well as in response to sensory stimuli, attention and other higher brain functions such as planning, reward and regulation of executive functions [128,129,131].

*FGF1* belongs to the *FGF* family, which is involved in several pathological conditions such as metabolic disorders, participates in the regulation of physiological processes such as the development, angiogenesis, adipogenesis and neurogenesis of the CNS, performs basic functions during embryonic development [134] and is involved in processes such as proliferation, adhesion, differentiation, survival, apoptosis, neuronal plasticity and cell motility [135]. *FGF1* signaling is required during the formation of neural plaque because it modulates the growth and pattern of specific brain structures (i.e., dorsolateral prefrontal cortex and the anterior cingulate cortex, which is involved in some components of working memory, especially central executive and episodic buffer) [136,137,138,139]. Furthermore, *FGF1* plays an important role in sensory development [137,140], specifically in the modeling of the bipotential optical vesicle [141,142,143], and improves sensory responses, especially in discrimination, comparison and location tasks [144].

*ADGRL3* (previously known as *LPHN3*), on the other hand, is a member of the latrophilin subfamily of G-protein coupled receptors and is highly expressed in the brain, particularly in the amygdala, the caudate and pontine nucleus, and cerebellum [58,59,60,86,93,145]. *ADGRL3* plays an important role in cellular adhesion and signal transduction, and is also expressed in the cornea [146] and is associated with alterations in the neuronal activities in visual tasks (i.e., Go/No-Go tasks) [147]. Latrophilins are relevant for neuronal development and brain functions [148]. Furthermore, *ADGRL3* has been shown to interact with *DRD4* (i.e., dysfunction and signaling in *DRD4* are mediated by the action of *ADGRL3* [86,149]), affecting the development of dopaminergic neurons [150].

We identified that variant *ADGRL3*-rs1565902 is associated with the arithmetic subtest (Table 2), which would require high mental effort to perform mental calculations and hence implicate activity of the central executive and episodic buffer of working memory. Numeric sense has been considered a non-verbal skill that involves spatial relationships between numbers at the mental level [151]. This information is represented and processed by regions of the bilateral lobes (i.e., inferior parietal lobe and precuneus), frontal–striatal and mesial temporal activation, and by the prefrontal (i.e., superior and medial frontal gyri) and inferior frontal and intraparietal sulcus [151,152,153], where *ADGLR3* is expressed [89]. As calculation tasks become more complex, there is a greater activation of the frontal region and cortical regions that underlie magnitude processing [154,155], which are important for numerical, visual and attentional processing [156,157,158], and the representation of the semantic aspect of quantity [159], which is directly related to working memory, including visual processing, speech understanding and episodic memory [160,161]. Finding that variants within *ADGRL3* are associated with ADHD cognitive endophenotypes may help to determine the nature of the cognitive alterations interacting with the genetic risk of ADHD [16,17,49,162].

We found that marker *FGF1*-rs2282794 is associated with the object assembly and analogies endophenotype, which is correlated to working memory components that have connections with perceptual aspects established as dual information processing (Table 2) [163]. *FGF1*, involved in the development of the eye and the demarcation of the neural retina, the pigmented epithelium [143], lens formation [143,164] and axonal growth [142,165] as well as in neuronal protection and survival [166,167], deserves greater attention due to the interruptions in the frontoparietal, dorsal attentional, motor and visual networks [168], which may lead to significant reductions in the volume of gray matter in the early visual cortex and specialized cortical areas for the identification of visual stimuli based on color, orientation and other aspects of shape [169,170,171]. In addition, some studies report the deactivation of the parietal and occipital regions during spatial tasks [109,172]. Such deviations are directly related to difficulties in visual–spatial intelligence, where the ability to analyze and synthesize abstract stimuli and establish relationships between parts and non-verbal reasoning, evident in the object assembly tests [173] as well as in the grouping of information for the formation of concepts, is a voluntary propositional process according to a series of inferential models of visual processing (i.e., analogies test) [174].

Marker *DRD4*-rs916457 is associated with the total digits and incomplete figures endophenotypes (Table 2) and expressed in key brain regions (Supplementary Figure S1). *DRD4* is highly expressed in the fronto-subcortical system in the hypothalamus, thalamus, olfactory bulb and hippocampus, which are part of the limbic system [130], as well as in cortical regions, the frontal cortex, occipital lobes and the cerebellum [35]. An imbalance of different dopaminergic transmission modes may be related to ADHD symptomatology [175]. Variants in *DRD4* are also associated with perceptual organization [176], which is related to the overlap between perception and visual memory that leads to a process of continuous perceptual alternation, where the brain must select a new interpretation in each repetition of the same stimulus by simultaneous and/or alternating action [177], suggesting a multiplicity of steps in this process occurring in hierarchically organized regions in the cortex. Thus, early visual areas register basic characteristics, and the superior areas unite them in objects and select the most relevant [178].

Despite our encouraging results, some limitations are to be acknowledged. First, the lack of pupil measurements in individuals of our cohort to determine the pupil diameter and rule out possible eye alterations that may impact perceptual organization is a limitation. Second, genotyped variants are localized in intronic regions. Although variants found to be in linkage and association give important insight into the neurobiological aspects of perceptual organization endophenotypes in our cohort, they may not necessarily be causal variants. In this sense, in silico and animal models may help to elucidate the role of such genetic variation at the protein level and how such changes may impact ADHD susceptibility, severity and long-term outcome. Third, only a few SNPs were available, which restricted the identification of potential associations in other genes of interest. This is a common problem, especially in understudied populations, such as the Caribbean community in Colombia [50,51,52].

In summary, our findings suggest that variants in *DRD4*, *ADGLR3* and *FGF1* are associated with ADHD endophenotypes related to perceptual organization and, as such, may constitute a new explanatory view of ADHD considering that people with the disorder present alterations in visual and speech perception, which are also determinants of symptom severity [116]. We also confirm the role of genes highly expressed in key regions of the brain related to attention and neurocognitive activity in affecting the metabolism of the neuronal circuits involved in ADHD in this Caribbean community. To the best of our knowledge, only a few studies have shown the association of marker *FGF1*-rs2282794 with ADHD and ADHD endophenotypes [17,52]. Finding this marker is also associated with endophenotypes of working memory and perceptual organization in our cohort (Table 2) gives supporting evidence about the role of *FGF1* as a potential candidate for ADHD. In neurosciences and neuropsychology, our results could contribute to the design, refinement and establishment of a theoretical construct of perceptual organization as a cognitive dimension in ADHD, supported by neurobiological evidence [179]. However, we are aware that replication in a population with different genetic backgrounds is needed. Future studies could greatly benefit from the use of high-throughput genotyping/sequencing for the identification of putative causal variants underpinning perceptual organization in ADHD, leading to the identification of genetic profiles better responding to specific ADHD treatments and the development of translational medicine approaches [180,181]. Furthermore, these variants could also be used for developing predictive models, based on machine learning and artificial intelligence [182,183,184], for ADHD diagnosis and the identification of severe ADHD cases [97,115,120,185] in the clinical setting.

## Figures and Tables

**Table 1:** ADHD cognitive endophenotypes in 408 individuals belonging to 113 nuclear families from Barranquilla, Colombia.

Coding *^a^*	Task	Affected (*n* = 236)	Unaffected (*n* = 172)	*d*	*P*	Heritability	
						*h*^2^ (SE)	*p*
	Mental Control	Mean (SD)	Mean (SD)				
T4	Numbers from 20 to 1 (Score)	2.13 (0.99)	2.55 (0.7)	−0.483	**0.034**	0.351 (0.138)	**0.006**
	**Semantic Verbal Fluency**						
T32	Token Test 36/36	31.36 (3.8)	33.51 (2.68)	−0.637	**0.001**	0.355 (0.124)	**0.002**
	**WISC-III and WAIS-III subtests**						
T42	Digit span total—Forward	6.84 (1.73)	7.8 (1.92)	−0.526	**3.7 × 10^−4^**	0.492 (0.107)	**1.0 × 10^−5^**
T43	Digit span total—Backward	4.53 (1.88)	5.24 (1.87)	−0.375	**0.001**	0.171 (0.102)	**0.048**
T44	Total punctuation (forward and backward)	11.32 (3.06)	13.12 (3.33)	−0.564	**1.6 × 10^−5^**	0.416 (0.109)	**6.8 × 10^−5^**
T45	Vocabulary	28.28 (10.63)	35.51 (10.99)	−0.670	**0.005**	0.452 (0.126)	**1.7 × 10^−4^**
T46	Comprehension	17.75 (6.27)	21.01 (5.88)	−0.533	**0.019**	0.210 (0.107)	**0.025**
T47	Arithmetic	12.94 (4.52)	12.87 (3.87)	0.016	**0.007**	0.365 (0.116)	**0.001**
T48	Similarities (analogies)	16.16 (6.98)	20.55 (5.89)	−0.671	**0.002**	0.366 (0.130)	**0.003**
T49	Figure completion	18.81 (4.86)	20.58 (3.45)	−0.410	**0.036**	0.235 (0.133)	**0.039**
T52	Object assembly	25.56 (8.8)	29.92 (9.13)	−0.488	**0.012**	0.323 (0.132)	**0.007**

*^a^* Refers to clinical variables/tasks in Cervantes-Henriquez et al. [49]. *d* = Cohen’s effect size; *h*^2^ = heritability estimated value. *p*-values < 0.05 are shown in bold. WISC-III = Wechsler Intelligence Scale for Children, 3rd edition; WAIS-III = Wechsler Adult Intelligence Scale, 3rd edition. A Logistic Regression for predicting ADHD diagnosis using these endophenotypes of working memory and perceptual organization led to an accuracy of 73% (95%CI = 68.4–77.2). Modified from Cervantes-Henriquez et al. [49].

**Table 2:** Results of the FBAT on ADHD endophenotypes in 113 nuclear families from an African-descent community.

Coding *^a^*	Chr	Marker	Gene	Position *^b^*	FBAT Results					
					Allele	Cohort	*P*_FBAT_ (NIF)			
						Frequency	Additive	Dominant	Recessive	HA
T44	11	rs916457	*DRD4*	637,014	T	0.050	0.026 (27)	0.025 (27)		
					C	0.950			0.025 (27)	
T46	4	rs10001410	*ADGRL3*	62,474,229	A	0.327		0.047 (54)		
					C	0.673			0.047 (54)	
T47	4	rs1565902	*ADGRL3*	62,408,620	C	0.495	**0.014** (65)			
					T	0.505	**0.014** (65)			
	5	rs2282794	*FGF1*	141,981,709	G	0.542		0.041 (32)		
					A	0.458			0.041 (32)	
T48	5	rs2282794	*FGF1*	141,981,709	G	0.542	**0.004** (64)	**1.9 × 10^−4^** (32)		
					A	0.458	**0.004** (64)		**1.9 × 10^−4^** (32)	
T49	11	rs916457	*DRD4*	637,014	C	0.950	**0.005** (27)		**0.005** (27)	
					T	0.050	**0.005** (27)	**0.005** (27)		
	5	rs2282794	*FGF1*	141,981,709	G	0.542		**0.006** (32)		
					A	0.458			**0.006** (32)	
T52	5	rs2282794	*FGF1*	141,981,709	G	0.542				**0.005** (64)
					A	0.458				**0.005** (64)

*^a^* Refers to subtests of the Wechsler Intelligence Scale for Children, 3rd Edition (WISC-III), and the Wechsler Adult Intelligence Scale, 3rd Edition (WAIS-III) batteries used Cervantes-Henriquez et al. [49]. T44: Total punctuation of the digit span (forward and backward); T46: Comprehension; T47: Arithmetic; T48: Similarities (analogies); T49: Figure completion and T52: Object assembly. See Table 1 for more details. *^b^* UCSC GRCh37/hg19 coordinates. Chr: Chromosome; HA: Heterozygous advantage; NIF: Number of informative families; FBAT: Family-based association test; *P*_FBAT_: *p*-value from the FBAT. For interpretation purposes, *p*-values in bold are statistically significant at 5% after correction for multiple testing using Bonferroni’s method.

## Supplementary Materials

The following are available online at https://www.mdpi.com/article/10.3390/brainsci11070854/s1, Figure S1: Multi-tissue eQTL comparison for *DRD4*-rs916457 according to GTExPortal (https://gtexportal.org/home/snp/rs916457); Table S1: Single nucleotide polymorphisms (SNPs) genotyped in 386 individuals belonging to 113 nuclear families from Barranquilla, Colombia.
