# Supplementary material for: ADGRL3, FGF1 and DRD4: Linkage and Association with Working Memory and Perceptual Organization Candidate Endophenotypes in ADHD
Source: Brain Sci. 2021 Jun 26;11(7):854. doi: 10.3390/brainsci11070854 (PMC8301925; doi:10.3390/brainsci11070854)
Supplement: Supplementary file 1 [file brainsci-11-00854-s001.zip › brainsci-1233030-supplementary.pdf]

brain sciences

MDPI

Supplementary Material

ADGRL3, FGF1 and *DRD4*: Linkage and Association to Working Memory and Perceptual Organization Candidate Endophenotypes in ADHD

Martha L. Cervantes-Henriquez [1,2,*,#], Johan E. Acosta-López [1], Mostapha Ahmad [1], Manuel Sánchez-Rojas [1], Giomar Jiménez-Figueroa [1], Wilmar Pineda-Alhucema [1], Martha L. Martinez-Banfi [1], Luz M. Noguera-Machacón [1], Elsy Mejía-Segura [1], Moisés De La Hoz [1], Mauricio Arcos-Holzinger [3], David A. Pineda [4], Pedro J. Puentes-Rozo [5,*], Mauricio Arcos-Burgos [3,*] and Jorge I. Vélez [2,*,#]

[1] Universidad Simón Bolívar, Facultad de Ciencias Jurídicas y Sociales, Barranquilla, Colombia, 080005; cervantesmh@unisimonbolivar.edu.co (M.L.C.H), jacosta@unisimonbolivar.edu.co (J.E.A.L), mostapha.ahmad@unisimonbolivar.edu.co (M.A), sanchezr@@unisimonbolivar.edu.co, gdjimenez@unisimonbolivar.edu.co (G.J.F), wpineda1@unisimonbolivar.edu.co (W.P.A), mmartinez108@unisimonbolivar.edu.co (M.L.M.B), lnoguera1@unisimonbolivar.edu.co (L.M.N.M), emejia18@unisimonbolivar.edu.co (E.M.S), mdelahoz48@unisimonbolivar.edu.co (M.D.H.M), ppuentes1@unisimonbolivar.edu.co (P.J.P.R)
[2] Universidad del Norte, Barranquilla, Colombia, 081007; jvelezv@uninorte.edu.co (J.I.V)
[3] Grupo de Investigación en Psiquiatría (GIPSI), Departamento de Psiquiatría, Instituto de Investigaciones Médicas, Facultad de Medicina, Universidad de Antioquia, Medellín, Colombia, 050010; mauricio.arcos@udea.edu.co (M.A.B), oscararcos98@gmail.com (M.A.H)
[4] Grupo de Neuropsicología y Conducta, Universidad de San Buenaventura, Medellín, Colombia, 050010; david.pineda@usbmed.edu.co (D.A.P)
[5] Grupo de Neurociencias del Caribe, Universidad del Atlántico, Barranquilla, Colombia, 081001.
* These authors contributed equally to this work.
# Correspondence: cervantesmh@unisimonbolivar.edu.co (M.L.C.H) and jvelezv@uninorte.edu.co (J.I.V.)

Table S1. Single nucleotide polymorphisms (SNPs) genotyped in 386 individuals belonging to 113 nuclear families from Barranquilla, Colombia.

Marker	Alleles	Chr	Position	Closest gene(s)	QC result	Observations
rs143020947	A/G	2	143,643,356	*KYNU*	NO PASS	Monomorphic
rs207462497	G/T	2	143,689,722	*KYNU*	NO PASS	Monomorphic
rs1565902	C/T	4	62,091,215	*ADGRL3*	PASS	-
rs10001410	A/C	4	62,156,824	*ADGRL3*	PASS	-
rs2122642	G/A	4	62,380,859	*ADGRL3*	PASS	-
rs6551660	C/G	4	62,390,745	*ADGRL3*	NO PASS	HW criterion
rs207464763	A/G	4	62,416,514	*ADGRL3*	NO PASS	Monomorphic
rs1510921	C/T	4	62,578,187	*ADGRL3*	NO PASS	HW criterion
rs6551678	A/G	4	62,705,646	*ADGRL3*	NO PASS	HW criterion
rs207464765	A/T	4	62,720,720	*ADGRL3*	NO PASS	Monomorphic
rs748519607	A/T	4	63,888,775	*ADGRL3*	NO PASS	HW criterion
rs28363183	G/A	5	1,444,398	*SCL6A3*	NO PASS	Monomorphic
rs2282794	A/G	5	141,961,893	*FGF1*	PASS	-
rs761010945	A/C	9	136,499,907	*DBH*	NO PASS	Monomorphic
rs569034387	C/G	9	136,500,791	*DBH*	NO PASS	Monomorphic
rs199926239	A/T	9	136,512,962	*DBH*	NO PASS	Monomorphic
rs916457	C/T	11	627,014	*DRD4*	PASS	-
rs1800443	G/T	11	629,830	*DRD4*	NO PASS	HW criterion
rs2236225	G/A	14	63,978,598	*MTHFD1*	NO PASS	Monomorphic
rs11568324	C/T	16	54,283,559	*SLC6A2*	NO PASS	MAF criterion
rs371002154	A/C	16	55,726,127	*SLC6A2*	NO PASS	Monomorphic
rs79777590	C/T	17	4,298,478	*UBE2G1/SPNS3*	NO PASS	Monomorphic
rs565399294	A/G	17	4,395,222	*SPNS3/SPNS2*	NO PASS	Monomorphic
rs2228130	C/T	17	7,345,715	*POLR2A*	NO PASS	MAF criterion
rs6108461	A/G	20	10,215,270	*SNAP25*	NO PASS	HW criterion
rs362990	A/T	20	10,224,221	*SNAP25*	PASS	-

[a] UCSC GRCh37/hg19 coordinates. Chr: Chromosome; QC: Quality control; HW: Hardy-Weinberg. Markers highlighted in **blue** passed quality control filters and were further included for analyses.

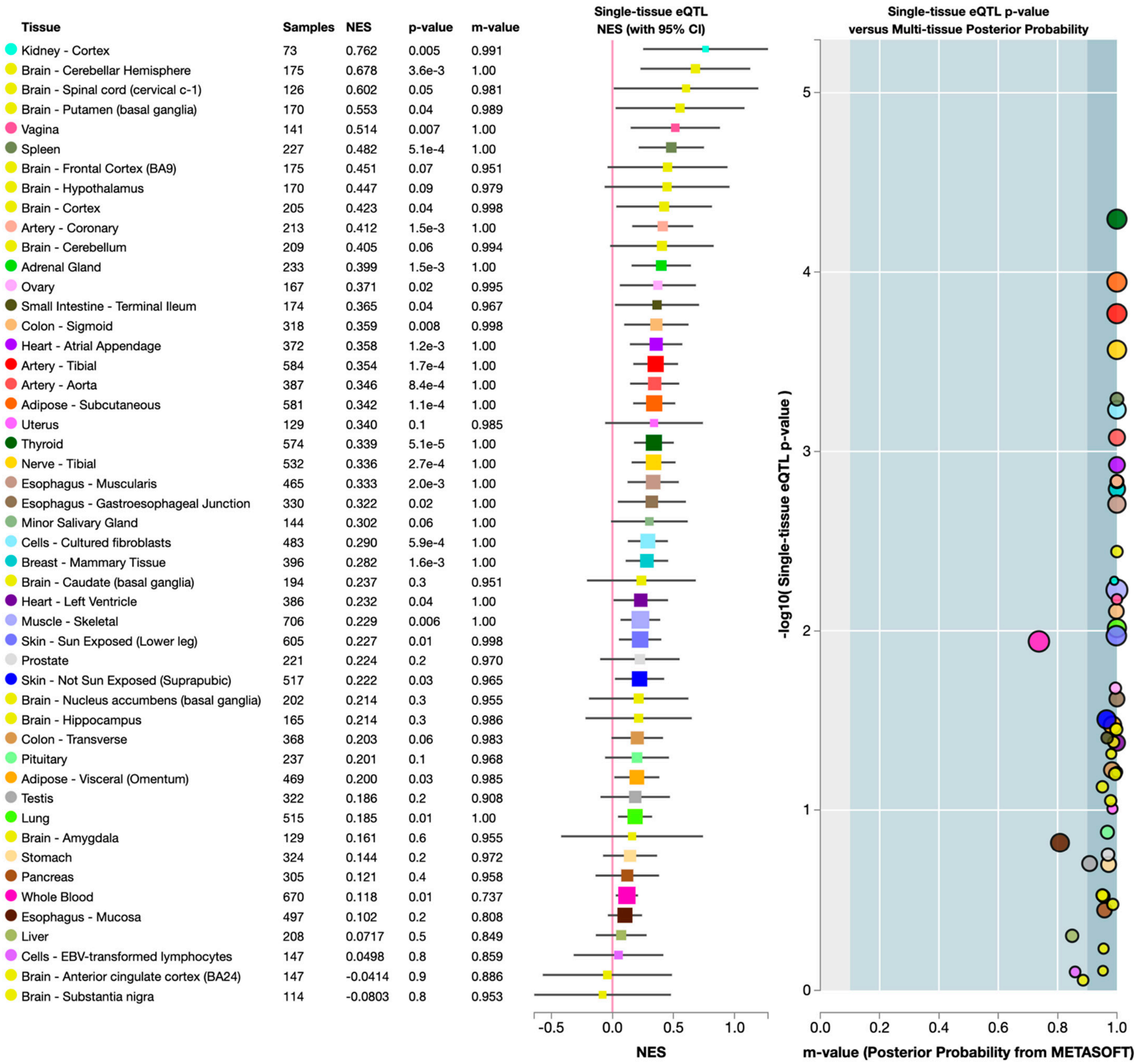

Tissue	Samples	NES	p-value	m-value
Kidney - Cortex	73	0.762	0.005	0.991
Brain - Cerebellar Hemisphere	175	0.678	3.6e-3	1.00
Brain - Spinal cord (cervical c-1)	126	0.602	0.05	0.981
Brain - Putamen (basal ganglia)	170	0.553	0.04	0.989
Vagina	141	0.514	0.007	1.00
Spleen	227	0.482	5.1e-4	1.00
Brain - Frontal Cortex (BA9)	175	0.451	0.07	0.951
Brain - Hypothalamus	170	0.447	0.09	0.979
Brain - Cortex	205	0.423	0.04	0.998
Artery - Coronary	213	0.412	1.5e-3	1.00
Brain - Cerebellum	209	0.405	0.06	0.994
Adrenal Gland	233	0.399	1.5e-3	1.00
Ovary	167	0.371	0.02	0.995
Small Intestine - Terminal Ileum	174	0.365	0.04	0.967
Colon - Sigmoid	318	0.359	0.008	0.998
Heart - Atrial Appendage	372	0.358	1.2e-3	1.00
Artery - Tibial	584	0.354	1.7e-4	1.00
Artery - Aorta	387	0.346	8.4e-4	1.00
Adipose - Subcutaneous	581	0.342	1.1e-4	1.00
Uterus	129	0.340	0.1	0.985
Thyroid	574	0.339	5.1e-5	1.00
Nerve - Tibial	532	0.336	2.7e-4	1.00
Esophagus - Muscularis	465	0.333	2.0e-3	1.00
Esophagus - Gastroesophageal Junction	330	0.322	0.02	1.00
Minor Salivary Gland	144	0.302	0.06	1.00
Cells - Cultured fibroblasts	483	0.290	5.9e-4	1.00
Breast - Mammary Tissue	396	0.282	1.6e-3	1.00
Brain - Caudate (basal ganglia)	194	0.237	0.3	0.951
Heart - Left Ventricle	386	0.232	0.04	1.00
Muscle - Skeletal	706	0.229	0.006	1.00
Skin - Sun Exposed (Lower leg)	605	0.227	0.01	0.998
Prostate	221	0.224	0.2	0.970
Skin - Not Sun Exposed (Suprapubic)	517	0.222	0.03	0.965
Brain - Nucleus accumbens (basal ganglia)	202	0.214	0.3	0.955
Brain - Hippocampus	165	0.214	0.3	0.986
Colon - Transverse	368	0.203	0.06	0.983
Pituitary	237	0.201	0.1	0.968
Adipose - Visceral (Omentum)	469	0.200	0.03	0.985
Testis	322	0.186	0.2	0.908
Lung	515	0.185	0.01	1.00
Brain - Amygdala	129	0.161	0.6	0.955
Stomach	324	0.144	0.2	0.972
Pancreas	305	0.121	0.4	0.958
Whole Blood	670	0.118	0.01	0.737
Esophagus - Mucosa	497	0.102	0.2	0.808
Liver	208	0.0717	0.5	0.849
Cells - EBV-transformed lymphocytes	147	0.0498	0.8	0.859
Brain - Anterior cingulate cortex (BA24)	147	-0.0414	0.9	0.886
Brain - Substantia nigra	114	-0.0803	0.8	0.953

Figure 1S. Multi-tissue eQTL Comparison for *DRD4*-rs916457 according to GTExPortal (https://gtexportal.org/home/snp/rs916457).
